# Microbial Degradation of Synthetic Biopolymers Waste

**DOI:** 10.3390/polym11061066

**Published:** 2019-06-20

**Authors:** Valentina Siracusa

**Affiliations:** Department of Chemical Science, University of Catania, Viale A. Doria 6, 95125 Catania, Italy; vsiracus@dmfci.unict.it; Tel.: +39-338-7275526

**Keywords:** biodegradable polymers, bioplastics, renewable resources, eco-friendly materials, microbial degradation

## Abstract

Over the last ten years, the demand of biodegradable polymers has grown at an annual rate of 20–30%. However, the market share is about less than 0.1% of the total plastic production due to their lower performances, higher price and limited legislative attention in respect to the standard materials. The biodegradability as a functional added property is often not completely perceived from the final consumers. However, the opportunity to use renewable resources and to reduce the dependency from petroleum resources could become an incentive to accelerate their future growth. Renewable raw materials, coming from industrial wastes such as oilseed crops, starch from cereals and potatoes, cellulose from straw and wood, etc., can be converted into chemical intermediates and polymers, in order to substitute fossil fuel feedstock. The introduction of these new products could represent a significant contribution to sustainable development. However, the use of renewable resources and the production of the bioplastics are no longer a guarantee for a minimal environmental impact. The production process as well as their technical performances and their ultimate disposal has to be carefully considered. Bioplastics are generally biodegradable, but the diffusion of the composting technology is a prerequisite for their development. Efforts are required at industry level in order to develop less expensive and high performance products, with minimal environmental impact technologies.

## 1. Introduction

**Figure 1:**
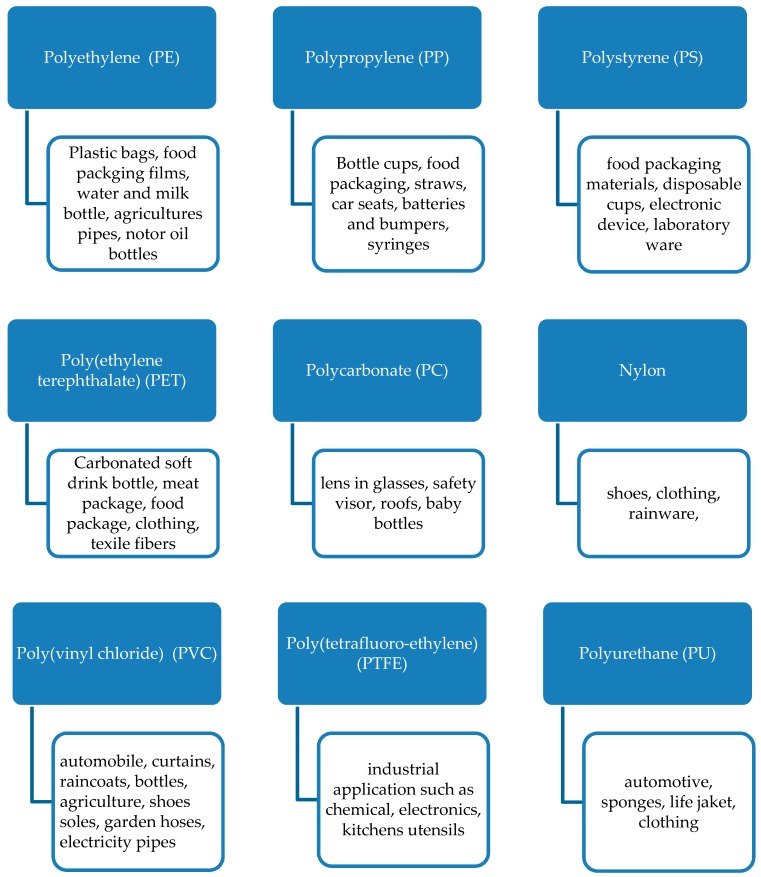
Synthetic plastics.

During the past 60 years, polymers materials, namely plastic, became the most attractive materials thanks to their low cost, ease of handling, lightness, high resistance to physical, mechanical and chemical aging and biological attack. The word plastic, which means “able to be molded into different shapes" (from the Greek word *plastikos*), comes rapidly and violently in the international dictionary. At the beginning, materials obtained from natural origin such as proteins and cellulose and their derivatives such as bone, wood, leather, etc. were firstly studied. Then, gradually, people learn how to modify them chemically, in order to enlarge the range of properties that could be considered. The chemical modification accompanied the production of an enormous kind of materials and thus, synthetic polymers started to substitute the natural materials in nearly completely every area, becoming an indispensable part of our life. The stability and durability of those materials were improved continuously, conferring them the synonym of materials resistant to environmental decay. At the beginning, their resistance to the degradation action was their strong point. Successively, their increasing presence into the living system becomes even more problematic. Surgery, pharmacology, agriculture, food and engineering are just some of the most involved areas where those materials are omnipresent. Used for a limited period of time, they became waste rapidly. At present, about 140 million tons/year of polymers are produced worldwide, the majority coming from petroleum resources. They are extensively used as packaging for food, pharmaceutical, cosmetics, detergents and chemicals [1]. About 30% of such production are utilized for packaging application, with about 90% only for food packaging application. Their use is always in expansion, at a rate of 12% per annum. This means that a remarkable amount of these materials are introduced in the ecosystem as industrial wastes. Owing to their even more visible presence in litter, plastics have gained the public as well as the media attention, more than any other solid waste. The most common synthetic plastics are reported in Figure 1, with the indication of their most frequent use.

The dramatic increase of their production, as well as their lack of biodegradability, focused the world’s attention on the pollution problem [2]. Plastics are disposed through landfilling, incineration and recycling. If improperly disposed, they become a significant source of environmental pollution, for centuries. If improperly burned, they produce persistent organic pollutants (POPs), such as furans and dioxins. If improperly recycled, they became further wastes, difficult to treat.

From 1980, plastics started to be designed in order to be susceptible of microbial attack and in the last 10–15 years several biobased and biodegradable materials have been studied. However, only few of them were industrially introduced into the market due to the high cost of production, still too expensive in proportion to the quantity of materials needed for each field of interest. This has led to an even more interest by the scientific communities to the developing of degradable polymers, which could be subject to biodegradation, photo-degradation, thermal degradation and/or environmental erosion mechanisms. Biobased polymers and biodegradable polymers are the candidates and, to produce them, it is becoming even more interesting to use alternative resources as raw materials. While synthetic biopolymer must be produced from renewable resources because they have to be biodegradable and compostable, bioplastics materials could also be obtained from other resources than renewable ones, because their biodegradable nature is directly correlated to their chemical structure rather than to their origin [3]. So, biodegradable plastic opened a new era for the waste management strategies, with the possibility to degrade them under environmental conditions or in municipal/industrial waste treatment units.

**Figure 2:**
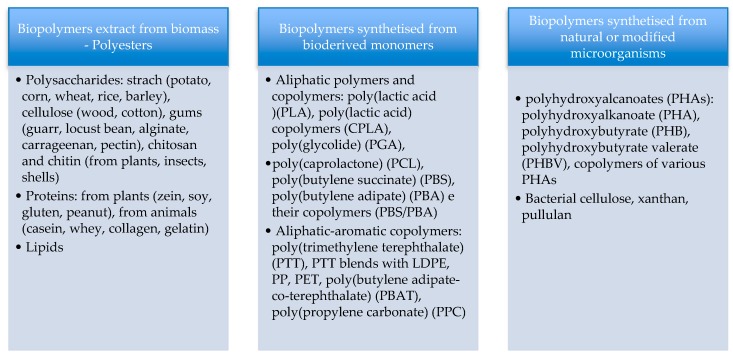
Biobased packaging materials.

The biodegradable polymers studied until now are reported in Figure 2. They are divided into three main groups, depending on the production process and on the sources [4].

As reported from Siracusa et al. [2], thanks to the economic process technology and easy production of the starting chemical molecules (monomers), polyesters are the favorite [5,6].

When a new biodegradable polymer is introduced into the market, two characteristics must be clarified: Biodegradability and biodegradation [7]. The first term is related to its chemical structure and so to the potentiality of such material to be degraded by biological attack. This characteristic is examined in the laboratory, following standardized tests. The second term is related to the process of degradation, that could happen if certain conditions are present, such as temperature, pH, moisture, etc. A polymer could be defined biodegradable but if the environmental conditions are not suitable, the polymer can show a very limited biodegradability. In order to assess the biodegradability, laboratory tests are performed exposing the polymers to microbial attack, in soil and in water. The degradation is measured as CO_2_ evolution or O_2_ consumption, both related to the mineralization process. The organic carbon is transformed in inorganic carbon (CO_2_) as a consequence of microbial respiration. Of course, not all biopolymers could be defined as biodegradable. Their behavior on the degradation process depends on their structure [8]. Some polymers are resistant to biological degradation because their carbon linkages cannot be broken from enzymes and microorganisms. The hydrophobic character limits further the enzyme activity together with other factors like low surface area, high molecular weight and crystallinity. Of course, all such characteristics are useful to obtain stable and durable polymers. In the past two decades, industries and researches have been challenged to develop biodegradable polymers, easy to process, with superior performance properties and competitive with conventional polymers coming from petroleum resources. This chapter will briefly outline the characteristics and microbial performance, positive and negative, of synthetic biodegradable polymers commercialized and studied to date. 

## 2. Microbial Degradation of Synthetic Biopolymer Wastes 

The term biodegradation is used to describe the mechanism that leads to a general damage of a material and it is often related to terms such as ecology, waste management, environment and of course plastic, due to their long life duration. Another term related to them is biomineralization, related to the conversion of organic matter into minerals. Natural and synthetic plastic could biodegrade aerobically (with oxygen) in wild nature, anaerobically (without oxygen) in sediments and landfills and partially aerobically/anaerobically in compost and soil. During the aerobic biodegradation, carbon dioxide and water are produced, while during the anaerobic biodegradation, water and methane is produced. The mineralization instead requires different microorganisms able to break the polymers backbone into its constituents (monomers), able to use these monomers and excreting by-products waste compounds and able to use the excreted wastes. In the presence of microorganisms, such as bacteria and fungi, the macromolecular chains are broken, starting the biodegradation process. This process proceeds under different conditions because the microorganisms involved are different from each other and are particularly active in the soil. Further, depending on the polymer characteristics such as chain mobility, crystallinity, molecular weight, type of functional groups, type of substituents present in the polymer structure, presence of plasticizers and/or additives, the degradation process could be deeply influenced. Two key steps are involved in the degradation process: (i) The polymer cleavage backbone, named depolimerization, where hydrolysis and oxidation are the main responsible, together with the extracellular enzymes, responsible for the cleavage on internal linkage (endo-action) and on the terminal polymer molecules (exo-action); (ii) the mineralization, which occurs inside the cell responsible for microbial degradation. In this manner, during the degradation process, the polymers are firstly transformed in its monomers that are progressively mineralized [1,9]. The microorganisms attack the end of the macromolecules. Considering that the number of ends is inversely proportional to the molecular weight of such polymers, to make plastic degradable is necessary to reduce their dimension and increase their surface area. Chain branching and crystallinity are further obstacles to this process. Synthetic biodegradable polymers degrade by the enzyme-catalyzed process, generally occurring in aqueous media. The most important group of synthetic biodegradable polymers is polyesters with hydrolyzable linkages along the polymer backbone. PLA, PCL, PBS, PBSA, are only few of them. Among them PLA and its copolymers with other polymers such as PCL, are the most commercialized to date. Several information about those polymers is present in literature [10,11]. During the non-enzymatic degradation process, by random hydrolytic chain scission of ester linkage, small molecules are produced. This process is enhanced by higher degradation temperature (from 40 to 60 °C). When the molecular weight approaches the value of 10,000, microorganisms start their digest process of the lactic acid oligomers, producing carbon dioxide and water. The amorphous PLA is biodegraded by proteinase enzyme while the microbial degradation of oligomers and polymers of PLA is not observed at an appreciable rate except when it is copolymerized with PCL. 

Aliphatic-aromatic polyesters biodegradable materials, containing hetero-atoms in their main chain, are the most interesting polymer thanks to their better performance in respect to the aliphatic ones (such as PLA). By combining the excellent performance of the aromatic polyesters with the biodegradability behavior of the aliphatic polyester, a number of copolymers have been developed. For technical application, thanks to the low cost and well-defined technique of production, PET and Polybutylene terephthalate (PBT) are widely used, but from the biodegradable point of view they are inert. Including in their structure several elements susceptible of biodegradability, gives the possibility to produce low cost and very high performance materials. This attempt was achieved by introducing an aliphatic acid component in the aromatic polyester chain. Between several chemical bonds, ether-bonds, amide-bonds and ester bonds are susceptible of hydrolytic attack, leading to a molecular weight reduction. These small molecules become water-soluble intermediates, able to penetrate the biological membrane to be mineralized. The cleavage backbone can be catalyzed by enzyme, connected to microbial degradation. As reported from Müller [12], during the last few years several aliphatic-aromatic copolyesters were synthesized as degradable materials. They are formed by PET, PBT, Polyethylene isophthalate (PEIT), Polypropylene terephthalate (PPT), poly(1,2-propanediyl phthalate) (PPP) and Poly(hexanemethylene terephthalate) (PHT) as aromatic moiety and oxyethylene diol, oxybutylene diol, ε-caprolactone, glycolic acid, oxalic acid, adipic acid, l-lactic acid, sebacic acid, succinic acid, fumaric acid, ethylene glycol, 1,12-dodecane dicarboxylic acid, 1,4-cyclohexane dimethanol as aliphatic moiety. From the results reported it was observed that the degradation rate decreases by increasing the aromatic fraction, which consequently increases the melting point. Random copolymers of PBT, with adipic acid as aliphatic moiety (PBTA), was considered the most promising biodegradable material for technical application, both for the low price monomers as well as for the degradation behavior. The degradation process is principally caused by microorganisms that excretes enzymes into the environment and that attacks the polymer surface, cleaves the polymer chains, starting their water solubility and soil interaction. Despite microorganisms being fundamental for the entire degradation mechanism, it can be also of abiotic nature, such as the one followed by PLA polymer, hydrolyzed under natural conditions by non-enzymatically process. The small oligomers and monomers formed during the biodegradation process are metabolized by microorganisms, giving rise to chemicals such as CO_2_, H_2_O and CH_4_, that becomes part of the natural cycles.

The most important parameter controlling the rate of the biodegradation process of aliphatic-aromatic copolyesters is the temperature difference between the melting point of the materials and the degradation temperature. The temperature is related to the polymer chain flexibility and consequently to the mobility of such chains to fit into the enzymes active sites. The flexibility is essential also for the degradation behavior. Longer aliphatic chains exhibit higher degradation rate. Aromatic rings instead act as obstacles to the degradation process, due to the steric hindrance of the enzymic to attack the ester bonds. It was observed that block copolyesters are more resistant to biodegradation than alternating copolyesters of the same overall composition, due to a very different melting point.

The degradation of such copolymers during composting has been largely discussed due to the fact that it represents the major way of polymer waste degradation. Under thermophilic condition, actinomycetes are the principal responsible copolymer degradation. Fungi were not found to play a relevant role in the degradation because they only grow at a temperature less than 50 °C. Many lipases are instead responsible for the depolymerization of synthetic polyesters. Compared to compost, the degradation in soil as well as in a liquid environment is slower, due to the lower temperature, to the variability of the environmental parameters such as humidity, pH, temperatures and soil and water composition. Further, in an anaerobic environment, such copolyesters are stable. 

## 3. Factors Affecting Degradation Process of Synthetic Biopolymer Wastes

The degradation of synthetic plastic is a slow process that involves several environmental factors. In general, light, heat, moisture, chemical and biological attack could provoke physical and chemical change in polymers, leading to a molecular chain degradation. According to the definition reported in literature [13], the biodegradation of a polymer is primarily induced by the action of different microorganisms but several others factors can contribute to the degradation process. Irradiation, thermal degradation and chemical hydrolysis could be some of the non-biotic effects to be considered. By absorbing solar radiation such as UVA (315–400 nm) and UVB (295–315 nm) responsible for photo-degradation process (photolysis, photo-oxidation), visible radiation (400–700 nm) responsible for the heating degradation and infrared radiation (760–2500 nm) responsible for thermal oxidation, polymers start to change their properties. Physical and optical property as well as molecular weight, ductility, embrittlement, chalking, cracking, color changes are just some of the observed modification of the polymers properties, allowing them to biodegrade. Despite the water being the basic environment required by the microorganisms to start their activities, a different type of non-liquid environment such as soil, compost or solid surface could be enough for the biological activity. In general two ambient are considered: A liquid environment and soil.

### 3.1. Biodegradation in Liquid Environment

In general, any biological process is related to the presence of water so, all biological degradation occurs in a liquid environment. Liquid environment means sweet water (lakes, river), marine environment (sea) but also waste water treatments (aerobic/anaerobic) and laboratories liquid broth. The liquid microbial population as well as the enzymes and the intermediate concentration plays a key role in the biodegradation process. In the easier definition, the aqueous environment surrounding the cell membrane is the perfect media for the diffusion of nutrients, excreted enzymes and metabolic products. As reported from Müller [14], soil humidity of 50–60% is optimal for aerobic biological processes. In this chapter, all the Standards test methods for low molecular weight chemicals available in this field and for biodegradable plastic are reported. 

The most studied biopolymers in real liquid environment are the polyhydroxyalkanoates such as PHB and PHBV and several polyesters such as PCL and PLA [14]. For PHAs polymers and copolymers, tests were performed in seawater at temperature range of 14–25 °C, in tropical coast water in the range of 25–32 °C, in sweet water in the range 6–8 °C, observing in all case a high degree of degradation, within at maximum 400 days. A complete defragmentation was observed for PCL in sea water at temperatures between 9–21 °C, within 56 days, by chemical hydrolysis and enzymatic surface degradation. In waste water plant, the degradation was faster for PHAc polymers while it was not influent for PCL polymer. However, it must be pointed out that the degradation test performed in real environment presents several limitations. Temperature and water quality can vary during the test period, making the experiments difficult to monitor. To overcome those problems, degradation in the laboratory test simulating a real aquatic environment is more suitable. The test could be performed in aerobic liquid environment and in anaerobic liquid environment. PHB, PCL and PLA were analyzed in a laboratory test with seawater, at 25 °C. While PHB and PCL showed faster degradation, PLA did not show any change. This behavior could be ascribed to the different degradation mechanisms. PHB and PCL are attacked by enzymes while PLA degrade by non-enzymatic catalyzed hydrolysis mechanisms, strongly depending from the temperature. While PHB degrade faster in both seawater and in sweet water, PCL degrade faster only in sweetened water. PBS shows the same rate of degradation as the PHBV copolymer, while PBA degrade slower in the same condition. Nevertheless, PBS exhibits no microbial degradation in seawater. This different behavior could be attributed to the unusual ability of the microorganisms to attack natural and synthetic polyesters. For PHAs polymers and copolymers, obtained from natural organisms that utilize carbon and energy to degrade, the degradation process is more easy and natural. For synthetic biopolyesters, the number of organisms able to attach their chemical structure is limited. As a consequence, the probability of degradation of such synthetic polyesters depends on the particular environment and microbial population. In the absence of oxygen, fewer enzymes are present and the growth of anaerobic microorganisms is slower. The degradation of PHB and PHBV was much slower but very similar. 

### 3.2. Biodegradation in Soil

While plastic material could be safe before biodegradation, after degradation its chemical composition could change, and consequently become unsafe and toxic for the environment. The biodegradation of polymers could create molecules that are accumulated in the environment, temporarily or permanently. Oligomers, monomers and metabolic intermediates can interact with living organisms in the soil, with negative effect on the environment. Consequently, becomes strictly important to understand the ecotoxicity of biodegradable polymers in soil. In general, this toxicity could be due to four reasons. The first one is associated with the possibility that small molecules such as additives and/or unreacted monomers, could migrate into the soil, causing temporarily or permanently toxic effect, according to their chemical-physical and mechanical stability. The second reason is due to the ageing effect such as sunlight, temperature, pH, acidic or basic surroundings, etc., which are able to degrade the polymer backbone into small toxic molecules, persistent in the environment. The third reason is associated with the biodegradation process, affecting the natural organic substrate. Consequently, toxic compost becomes not suitable for agriculture use due to its not right degree of maturation that could depress the plant growth. This effect could be permanent or not in the environment. The fourth reason is associated with the accumulation into the soil of toxic residues and intermediates coming from the biodegradation process of the polymers. 

When studying the biodegradation in soil, it must be considered that two kinds of situations could be present: Biodegradable polymers which are disposed by the composting process (littering) and biodegradable polymers which are intended to biodegrade directly into the soil, such as the materials used in agriculture (as for example for mulch, irrigation tubes, pots, etc.). Since the soil is used for producing food for human and animals, too much attention has to be paid in order to assess any negative and persistent influence of in situ plastic disposal. Another possibility must also be taken into consideration: The unintentionally presence of the polymers into the soil. If intentionally disposed, biodegradable polymers are utilized for producing compost. In this case, plastic must be susceptible to being reduced in particles less than 2 mm of dimension, in less than three months of composting [15]. If after this time, small pieces of plastic are persistent, they must complete their mineralization process together with the mature compost, just present in the environment. In general, the degradation time must be compatible with the crop cycle. If unintentionally disposed, the plastic littering problem is present. No matter if the polymers spread is biodegradable, the environmental problem is very serious. Undoubtedly, biodegradability is a great feature to solve the problem.

When considering the soil environment, it must be considered that several kinds of soil are present, which vary from place to place. The soil characteristics are strictly related to several uncontrolled parameters such as temperature, water content, chemical composition and pH, all related of course to the geographical factors. All those factors could be joined together in a different way, creating different environments. Consequently, the microbial biodegradation process can change from place to place, from season to season. The degradation process studied under composting condition is easier to treat because the variability of the compost with the environment is very low. This is a well-balanced industrial process, followed by any latitude, to obtain compost that must be chemically controlled, with earth smell and of course with marketable attributes. The biodegradation tests are easy to control due to the standardized environment. When studying the biodegradation in soil, the environmental factors could be very different. In this case, two factors and consequently two phases are present: Surface factors, induced by abiotic degradation, and underground factors. The first phase takes place under the action of climatic factors, and will be further fully described. The presence of water during the surface degradation process is of great importance. It can induce microbial growth on the plastic surface and loose of the mechanical performance owing to plastic leaching. The second phase takes place under the action of microorganisms, in the soil underground, where roots development takes place and where nutrients, water and microorganisms are available for starting the degradation process. Several factors could affect the polymer degradation that could be seen under the physical, chemical and biological point of view. First of all the structural properties of the soil are the particle size (soil texture) and the soil composition. The soil texture is related to the proportion of sand, silt and clay distribution. A harsh distribution can cause abrasion and so mechanical degradation. Further, the distribution of the aggregates could have a negative influence on the water and gas diffusion through the soil, and on the heat transfer. Those parameters have a great influence on the degradation plastic mechanisms and on the growth of the microorganisms responsible for the biodegradation. The soil temperature has a great influence also on the enzymic processes, on the polymer hydrolysis and on the polymer chain mobility. Enhancing the mobility, the contact between the polymer chain and the enzymic active size is facilitated, promoting chemical bond cleavage [16]. The presence of cations such as NH^4+^, K^+^, Mg^++^, Ca^++^ into the clay is important for the life of the microorganisms that use those chemicals as nutrient, serving as catalytic chemicals for facilitate the biodegradation process. The constitution of the organic matter (plant and animal residues) is important as a nutrient reserve, as water retainers and as soil structure improver. It comes from the mineralization process, and serves also to maintain an acceptable pH. It was seen that if the organic matter is added to the compost (10% by weight), the degradation process is accelerated, without any influence on the degradation pathway [15]. Water and gas are competitive for the aerobic/anaerobic degradation process. If the water content increases, the gas content (O_2_ and CO_2_) decreases, enhancing the anaerobic condition, less favorable for the biodegradation process. Water into the soil is important because it induces hydrolysis with consequently polymer bond cleavage and lower molecular weight formation molecules. Further, it facilitates the leaching of the additives present into the polymers, enhancing the brittleness. It has an important influence on the environmental pH, with consequently interference on the microrganisms life. As for example, an acidic soil has a negative effect on the bacteria development with a consequent decrease in soil mineralization and biodegradation. By neutralization performed with CaCO_3_ the rate of biodegradation increases. From a biological point of view, fungi are the main organisms present in the soil, active in both the decomposition and mineralization of different substances such as cellulose, lignin and chitin [15]. 

Not only the environment influences the polymer degradation. Other key aspects must be taken into consideration, related to the polymer chemical composition. This parameter is the most important because it governs the chemical and physical behavior of such materials. Many efforts have been made in order to correlate the polymer structure to their biodegradability performance. In particular, several authors studied new biodegradable materials synthesized by the copolymerization technique and their biodegradable character [17,18,19]. One of the most important factors is the physical state of the plastic because the surface must be susceptible of enzymes attack. Those enzymes have to break down the polymer chains into smaller molecules, easier to be assimilated from the microorganism present in the environment. In this case, the hydrophilic nature and the percent of crystallinity of the polymers play a key role. In general, high crystalline polymers are more difficult to degrade because is more easy for the enzymes to attack the amorphous phase. This is not true for all polymers. For example, starch materials and bacterial polyesters such as PHAs are rapidly hydrolyzed. The chemical characteristics are related to several factors: (i) The type of chemical linkage in the polymer backbone; (ii) type, position and numbers of pendant groups; (iii) type of end groups. If ester and amide groups are present, the enzymic attack is promoted. This behavior is not observed for aromatic compounds, polyamide and other polymers where the heteroatoms are present in the main chains. The stereochemistry of the polymer is also important because many enzymes involved in the degradation process are stereochemical selective. This obstacle could be passed through the use of microorganisms instead of enzymes. The molecular weight plays an important role. For polymers, a high molecular weight for high mechanical and gas barrier performances is requested but the depolymerization process is highly reduced. A critical lower limit must be reached before the degradation process will start. Blending with different polymers could affect the biodegradation behavior. From one side, this technique is utilized to enhance the mechanical performance as well as the moisture and gas barrier behavior of such materials but their resistance to microbial attack shows an increment. The biodegradability could be improved by grafting or crosslinking biodegradable polymers with non-biodegradable polymers, giving rise to biodegradable systems. 

## 4. Involvement of Enzymes in Degradation Process of Synthetic Biopolymer Wastes

**Figure 3:**
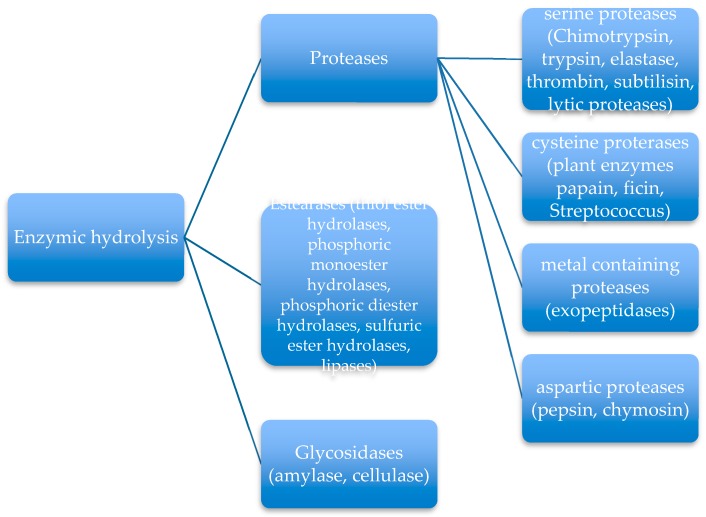
Enzymic hydrolysis.

Microbiological degradation of synthetic biopolymers takes place through the action of enzymes and acids and peroxides secretes from bacteria, fungi, yeast, etc. At the beginning, polymers must be reduced in a small dimension by the polymer chain cleavage, by extracellular enzymes. Then, the small oligomers and monomers formed are transported into the cell where they are mineralized. During this process several substances are produced such as CO_2_, CH_4_, N_2_, H_2_, water, salts, minerals, biomass and adenosine triphosphate (ATP). Independently from the environment and from the chemical nature of the polymers, enzymes are the principals responsible for the degradation process. Enzymes are biological catalysts that increase the rate of the chemical reaction otherwise not favorable in a natural environment. They are proteins like polypeptides with different molecular weight ranging from several thousand to several million g/mol. The presence of active sites, where the interaction between enzymes and substrate takes place, is the most important feature. Same enzymes are specific for a given substrate while others can be active for a series of substrates. The presence of other substances such as metal ions, vitamins, ATP and so on can assist their activity. Due to this great variety of enzymes, different kind of mechanisms of catalysis could be considered. The most important examples of polymer degradation through enzymes are enzymic hydrolysis and enzymic oxidation. For biopolymer degradation through hydrolysis, several enzymes are involved in the ester bond cleavage, as reported in Figure 3.

The different types of enzymes have different active sites. The Estearases are the most diffuse in nature and are involved in the splitting of the ester linkages. Lipase acts on the water-lipid interface and its mechanism of action is very difficult to explain. 

**Figure 4:**
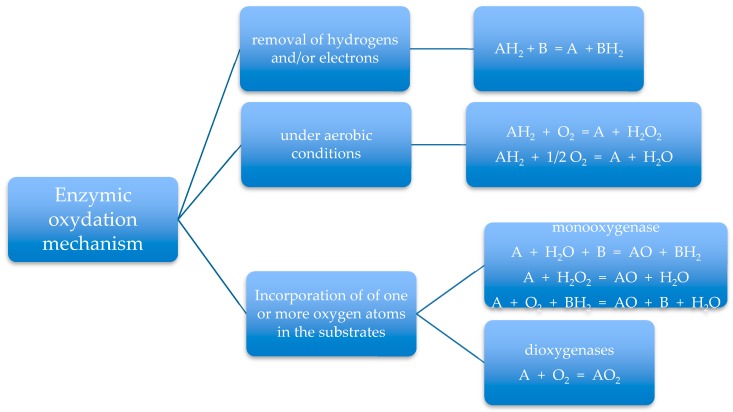
Enzymic oxidation mechanism. B, O_2_ and H_2_O_2_ are the electrons acceptors.

The mechanism of enzymic oxidation is summarized in Figure 4.

## 5. Mechanism and Pathways of Synthetic Biopolymer Degradation

As reported from Lucas et al. [20], the term biodegradation is associated with the biological activity. However, in the organic matter decomposition, both the biotic and abiotic factors must be considered. The abiotic degradation precedes the microbial assimilation, and must be absolutely considered when explaining the degradation mechanisms. 

**Figure 5:**
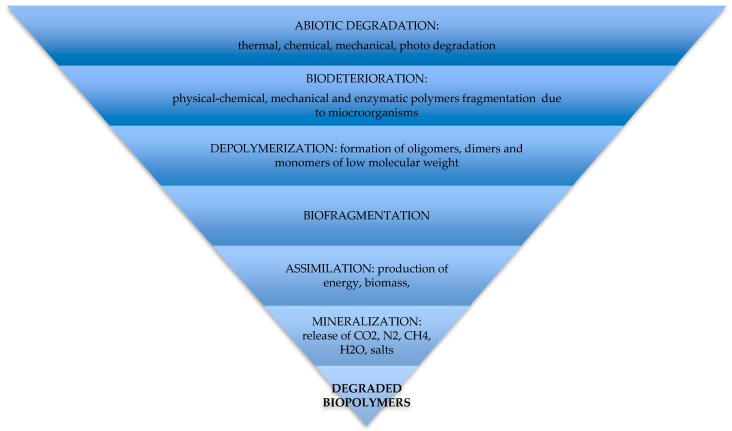
Biopolymers degradation steps.

The mechanism of the polymer biodegradation takes place in several stages and the process could be stopped at each stage. The degradation steps are reported in Figure 5.

### 5.1. Abiotic Degradation

External conditions such as weather, ageing, sunshine, soil burying, water could accelerate the degradation process. Polymers consequently undergo thermal, chemical, mechanical and photo degradation, becoming synergistic factors useful for the acceleration of the biodegradation process [21]. 

The temperature has a great influence on the macromolecular framework organization. Depending on the thermal parameters such as melting temperature (T_m_), glass transition temperature (T_g_), crystalline/amorphous ratio, connected to the chemical nature of the polymers, the influence of the temperature could act in a different way. Generally, the melting point of thermoplastics polymers is higher than the environmental temperature but some thermoplastics polymers have a melting temperature near the environmental conditions or composting temperature (as for example the PCL, with a T_m_ ≅ 60 °C). For polymers like biopolyesters (PLA, PCL, PBS, PHB, cellulose, etc.), with a semicrystalline structure, the structural change takes place around their glass transition temperatures, where the mobility of the macromolecular chains is modified. In the rubbery state (above T_g_), the amorphous organization of the polymers chains facilitates the degradation process, thanks to the lower rigidity of the material. In the glassy state (under T_g_), an increment of the temperature could promote the formation of additional crystallites (named spherulites). Interspherulitic cracks, with an increment of brittleness, could take place, facilitating consequently the degradation process [11,22,23,24]. Some polymers, such as the PBA, could present crystal polymorphism in the crystalline region, leading to a different interaction with the microorganisms. As reported from Gan et. al., the α-crystals (present above 32 °C) of PBA show a faster hydrolysis by the action of lipase *Pseudomonas* sp. than the β-crystals (present below 27 °C). In the range between 27 and 32 °C, both crystals are present, with a different response to the degradation process, depending on the surrounding temperature [25,26,27].

Chemical degradation can be due to the presence of atmospheric pollutants, agrochemical present into the soil, oxygen and water. Oxygen is the most powerful to create abiotic chemical degradation, exploited by the covalent bonds attack, producing free radicals. Of course, the polymer structure could greatly influence this process. As for example, unsaturated links or branched chains could be an obstacle or could promote the degradation process, leading to crosslinking reactions or chain scissions [28,29]. If in the polymers backbones are present hydrolyzable covalent bonds such as esters, ethers, anhydrides, amides, etc., the hydrolysis is another way of abiotic degradation process. Further, oxidative and hydrolytic degradation is promoted by the polymer amorphous phase, while the crystalline domains could represent an obstacle to the diffusion of oxygen and water, limiting the chemical degradation [20]. Water activity, pH, temperature and time must be controlled to promote the hydrolytic degradation. As for example, while the degradation of PLA, PCL and PPC is very slow in a neutral environment, in basic as well as acidic conditions, the degradability is very high [20,30,31]. The hydrolysis of the ester groups gives up the release of low molecular weight molecules, with consequently degradation of the polymer.

The mechanical damage due to compression, tension and shear forces can activate or accelerate the degradation process, at molecular level [29]. This abiotic parameter is not predominant but in combination with the temperature, chemicals and light it becomes synergic [21]. 

Photo-degradation induced by the light exposure of the polymer materials, is one of the most important abiotic parameters influencing the biodegradation process. It takes place by means of Norrish reactions, by photoionization (Norrish I) and chain scission (Norrish II) [32]. Tsuji et al. and Kijchavengkul et al. described the Norrish reactions for PLA and PCL and for PBAT, respectively [33,34]. 

### 5.2. Biodeterioration and Depolymerization

This step, focused on the biological aspect, is the subsequent consequence of the abiotic degradation. Once the polymer is fragmented, the microbial activity starts, on the surface of the material and inside the material [35,36]. Microorganisms can act as mechanical, chemical and enzymatic promoters. Depending on the polymer constitution, on the environmental conditions (temperature, humidity, weather, pollutants) and on the microorganism involved (bacteria, protozoa, algae, fungi, lichenaceae), the materials could be damaged in a different way. On the surface of the polymers a biofilm, of simple molecules, sources of carbon and nitrogen start to create, which could become nutrients for some microorganisms. Organic dyes, sulphur dioxide, aliphatic and aromatic hydrocarbons coming from urban air could represent further potential sources of nutrients for some microorganisms, enhancing the material degradation. Those microorganisms, attached on the polymer surface, act as glue layers, as a slime matter [37,38]. They penetrate into the polymer porous structure, altering the pores sizes and distribution, provoking crack and mechanical damage. The resistance and durability of the materials are consequently lowered. Further, this slime promotes the accumulation of pollutants, with a consequence development of microorganisms and biodeterioration acceleration [39]. As reported from Lucas et al. [20], each type of microbial flora developed into the materials contributes to the further chemical biodeterioration process. Any active materials produced by abiotic and/or biotic hydrolysis of polymers, such as nitrous, nitric, sulphuric, oxalic, citric, gluconic, glutaric, glyoxalic, oxaloacetic, fumaric, succinic, adipic, lactic acids, could promote the chemical biodeterioration. They induce water penetration inside the polymers pores, enhancing the hydrolysis process, accompanied by swelling, oligomers and monomers production. Those acids could also interact with the materials, enhancing surface erosion and could sequestrate cations such as Ca^++^, Si^4+^, giving rise to stable complexes [40,41]. 

The way of enzymatic degradation is more difficult. It requires the presence of several factors, as for example, cations and coenzymes synthesized by microorganisms [42,43].

### 5.3. Biofragmentation

In order to be assimilated from the microorganism, polymer materials need to be fragmented, that is reduced in small fragments of low molecular weight (oligomers and monomers). This process is indispensable because the high molecular weight macromolecules are unable to cross the cell wall and cytoplasmic membrane. The abiotic process, previously reported, provides the energy necessary to accomplish this scission thermally, chemically, mechanically and by photodegradation. The biological aspect instead requires the use of different microorganisms. As indicated by Lucas et al. [20], they secrete enzymes and free radicals to be used for cleaving the polymers backbone. While the enzyme involvement in the degradation mechanism was previously reported (§4), the same oxidation reaction could be catalyzed by free radicals produced from various enzymes. As indicated from Müller et al. [44], high crystalline polymers are not favorable to enzymatic attack because the path to the internal area is windy. It is so important that firstly the polymer is fragmented. Some soil decomposers such as brown-rot fungi, produce H_2_O_2_, high oxidative and reactive molecule. This molecule in the presence of ferrous atoms and acidic ambient, by Fenton reaction, produces free radicals OH, molecules extremely reactive but not specific. Fungi, in order to protect itself against degradation, produce small molecules that act as free radical transporters. They diffuse through the polymer matrix leading to chain fragmentation. 

### 5.4. Assimilation and Mineralization

The final stage, the assimilation, is the most problematic, due to the lack of validated techniques that will give a legal certification about the impact of those new materials into the environment. However validating the assimilation processes, such as mineralization, are important to guarantee the title of “friendly environmental materials”. In this step, a direct interaction between the polymer fragments and the microbial cells is evident. Microorganisms live thanks to the energy recovered from the polymers disintegration and using carbon, phosphorous, oxygen, sulphur as nutrients for their cell structure. Monomers coming from disintegration pass through the cellular membrane, are oxidized to adenosine triphosphate (ATP) and other elements useful for the cell structure formation. The energy required for supporting the cellular activity is obtained from three mechanisms: Aerobic respiration, anaerobic respiration and fermentation [20]. While mineral molecules released by microorganisms do not represent an eco-toxicological risk for the environment, the microbial organic molecules coming from the polymer disintegration could represent a real hazard at different level.

## 6. Microbial Toxicity of Synthetic Biopolymer Wastes and Degradation Products

**Figure 6:**
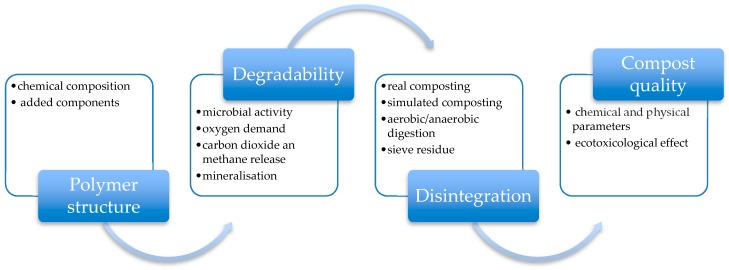
Degradation pathways of biodegradable polymer.

Until now, most of the efforts were oriented to synthesize and to develop novel biodegradable polymers and not much attention was given to the identification of the environmental requirements for those polymers. Further, plastic ends their life rapidly, becoming sooner waste or litter. Due to the fact that biodegradable materials are designed to be mineralized by the microorganisms present in the environment, the interaction with the ecosystem becomes very important. In this context, it is important to well understand how the by-products waste compounds could be identified and characterized in terms of their toxicology for the environment and for human health, considering further that different types of ecosystems are involved and must be considered. In the last few years, national and international standards were published in order to get an indication about the biodegradable polymers [45,46,47] and consequently the required test to establish their toxicity for the environment. In this contest, four steps have to be considered, as reported in Figure 6.

The first distinction is between biodegradation with subsequent organic recovery of the bio-wastes and the biodegradation process in the environment. The first process is due to the massive microorganisms work, supported by watering and aeration, and it is a combination of the thermo and mesophilic process. The second one is due to a less population of microorganisms, without support, at meso- or psychrophilic conditions [48]. Both are involved in the compost composition test, which could be exploited with the test polymer added and without any addition of polymers into the ecosystem. In such manner, the results coming from the chemical and physical analyses as well as the ecotoxicological effects on the compost could be compared with each other. In order to facilitate the interpretation of the results, it is more easily the determination of their influence on the seed germination rate or on the reduction of plant growth. Those parameters are connected to the compost quality and are independent from the chemical composition [49]. The introduction of toxic elements, such as for example heavy metals, halogenated or aromatic hydrocarbons, could contaminate the compost, being declassified to a second or third quality compost or becoming compost of limited use. However, the quality of the resulting compost should not be limited to chemical and physical data referred to the contents of nutrients and to the presence of pollutants, but also to the appearance of unknown metabolites. Eco-toxicological tests become mandatory, as described in the German and in the European standards [45,46,47]. Further, several books are present in literature and could be used to well study the ecotoxicology science [50,51,52,53]. When biodegradable polymers are introduced in the environment, certain microorganisms could be activated, which can be pathogenic to crop plants, soil and animals. If polymers containing elements other than carbon, oxygen and hydrogen, the effect of their biodegradation process must be seriously analyzed. Halogen and heavy metal introduced as pigments into the polymer structure may form by-products during the degradation process, that could cause serious environmental problems. However also pure hydrocarbons polymers could be incompatible with the environment, due to their incompatible chemical structure. The degree of polymerization is not only correlated to their biodegradability. It is necessary too that the polymer is available for extracellular bacterial enzymes. The presence of aromatic monomer in the polymeric chain plays a key role in the degree of biodegradability as well as in their availability [54,55]. Humic substances are formed, similar to soil and aquatic environment. These substances are organic molecules made of aliphatic and aromatic hydrocarbons, sugar and organic components containing elements such as nitrogen, sulfur, oxygen and phosphorus that may interact in several ways with the environment. Organic and inorganic pollutants present into the environment could consequently change their structure and properties. As reported from Fritz [48], humic substances can act in different ways: (i) They can change the cell membrane permeability; (ii) they can react by radical reaction, changing the chemical structure or organic molecules, that could be transformed in higher or lower toxic species; (iii) they can form chemical bonds with organic molecules, lowering their soil concentration on so decreasing their bioavailability. Further, humic substances can form chemical complexes with heavy metals like Cu, Sn, Ni and Zn, producing species soluble into the ground and surface water, increasing the toxicity or prevent their uptake into the cells [56,57]. 

## 7. Future Prospective

Biodegradability tests are necessary to estimate the environmental impact due to the polymer biodegradation. However, the realization of natural experiments in the laboratory scale is very difficult because of the non-reproducibility of the complex natural phenomena. The diversity of microbial communities, the catalytic pathway of nutrients transformation, the material chemical differences, etc., cannot be fully controlled and reproduced in vitro. The biodegradation mechanisms, described in literature [58,59,60,61] must be fully understood and accepted by all stakeholders. In this contest, a standardization work is required in order to give a uniform criteria for the classification of polymers that biodegrade in the environment, to assure the suitability of this new products. As reported by Degli Innocenti [15], the results obtained on the degradation study of traditional polymers have shown their recalcitrance to biodegradation in soil. Polyesters such as PLA, PHB, PCL, PBSA and its copolymers show a high variability of degradation in soil, depending on the selected test condition. Is important to design a unified methodological approach to generate reproducible data. Definitions and test methods must be built on two points: i) The realization of a standard procedure for the qualitative and quantitative evaluation of the biodegradability in order to avoid claims on the results and ii) definition of rules useful to prevent the accumulation of such materials into the environment. This latter point is related to a social expectation, where farmers, public authority and consumers are involved. The total biodegradability and the total absence of ecotoxicity are the main criteria required for the environmental protection. As for example, in agriculture is not possible to accumulate both plastic residues than recalcitrant molecule coming from the degradation process. The biodegradation of plastic must be fast and complete, to avoid soil contamination and to guarantee the crop cycle. The agricultural productivity should not be influenced by the eco-toxicity of the substances coming from the biodegradation of plastic materials. To avoid accumulation of non-biodegradable materials in the environment, the inherent biodegradability must be fully assessed, that is guaranteed by using standard tests methods. These methods are also needed to predict the durability and performances of such biodegradable polymers when in use, that is of commercial interest. A polymer could be suitable for agricultural application but not suitable for the environment and vice versa. A well balance between the two properties must be reached. 

Regarding the ecotoxicity of biodegradable polymers, a crucial factor is the interaction between organic and inorganic pollutants with humic substance. Polymers, degradation intermediates, residues, metabolites and environment could interact in a synergistic or antagonistic way. In order to avoid any adverse effect on the ecosystem, an extensive investigation is mandatory. 

## 8. Conclusions

Polymers must not have a negative influence on the environment in which they are applied. An ecosystem, solid or aquatic, must be protected in its natural balance. Actually, two approaches are considered to minimize their impact on the environment: 

The design of polymeric materials with long duration such as for example aeronautic device, construction, containers materials, etc., of great industrial interest; 

Innovative technologies, studied for the production of biodegradable polymers with short life, such as food packaging, agricultural mulches, medical devices, etc. 

The right balance between durable and biodegradable polymers is not easy to reach. The new materials are asked to be resistant during their use but biodegradable at the end of their life. One possibility could be the co-extrusion between natural and synthetic polymers. The synthetic polymers part could be recycled while biodegradable ones could be bio-deteriorated. When biodegradable polymers are released into the environment in vast amounts, different aspects have to be considered. Water resources, and not only drinking water resources, need to be protected among contamination. Soil must be preserved. The introduction of new artificial biodegradable materials together with the traditional bio-wastes could represent great risks in the production of compost if not analyzed and well-known substances are detected. Not all biodegradable polymers are suitable for composting process. Further, microorganism’s assimilation of oligomers and monomers, coming from the biodegradation process, is not yet totally proved. The importance of biodegradation is also related to the waste management, that is a balance between production and removal that must be controlled and regulated. Actually, unfortunately, the rate of plastic wastes production is higher than the disposal rate, but the increasing interest compared to the environmental preservation encourage researchers to develop new materials from renewable resources, as for example wastes. Materials obtained from wastes require a full base characterization in terms of mechanical, thermal, barriers and microstructure, as well as the possibility to control their recyclability instead of their biodegradability.
